# Angiopoietin‐like protein 8 expression and association with extracellular matrix metabolism and inflammation during intervertebral disc degeneration

**DOI:** 10.1111/jcmm.14488

**Published:** 2019-06-18

**Authors:** Zhiwei Liao, Xinghuo Wu, Yu Song, Rongjin Luo, Huipeng Yin, Shengfeng Zhan, Shuai Li, Kun Wang, Yukun Zhang, Cao Yang

**Affiliations:** ^1^ Department of Orthopaedics Union Hospital, Tongji Medical College, Huazhong University of Science and Technology Wuhan China

**Keywords:** angiopoietin‐like protein 8, extracellular matrix, inflammation, intervertebral disc degeneration, NF‐κB

## Abstract

Intervertebral disc degeneration (IDD) is considered the primary culprit for low back pain. Although the underlying mechanisms remain unknown, hyperactive catabolism of the extracellular matrix (ECM) and inflammation are suggested to play critical roles in IDD progression. This study was designed to elucidate the role of angiopoietin‐like protein 8 (ANGPTL8) in the progression of IDD, especially the relationship of ANGPTL8 with ECM metabolism and inflammation. A positive association between ANGPTL8 expression and degenerative grades of IDD was detected in the analysis of human nucleus pulposus tissue samples. Silencing of ANGPTL8 attenuated the degradation of the anabolic protein type collagen II, and reduced the expression of the catabolic proteins MMP3 and MMP9, and the inflammatory cytokine IL‐6 through inhibition of NF‐κB signalling activation. In addition, the effect of ANGPTL8 was evaluated in a rat model of puncture‐induced IDD. Based on the imaging results and histological examination in animal study, knockdown of ANGPTL8 was demonstrated to ameliorate the IDD progression. These results demonstrate the detrimental role of ANGPTL8 expression in the pathogenesis of IDD and may provide a new therapeutic target for IDD treatment.

## 1 INTRODUCTION

Intervertebral disc degeneration (IDD), which is a primary cause of low back pain (LBP), has brought about a global healthcare burden and great socioeconomic costs.^1^ Although factors such as gender, ageing, obesity and diabetes mellitus were identified to be associated with IDD, the pathological mechanisms involved in IDD progression remain unknown.^2^, ^3^, ^4^, ^5^ Current treatments for LBP include bed rest, anti‐inflammatory drugs and surgical intervention, such as discectomy and spinal fusion.^6^ However, these approaches are usually confined to relief of symptoms and symptoms are easily recurrent, and surgical intervention may result in restricted spine flexibility.^7^ Since the aetiology of IDD remains unclear, uncovering the pathogenesis of disc degeneration is crucial in establishing the therapeutic methods for IDD.

Intervertebral disc (IVD) is an avascular organ composed of the inner semi‐gelatinous nucleus pulposus (NP), the outer annulus fibrosus and cartilage endplate.^8^ The extracellular matrix (ECM) of the inner disc is constituted by collagens and proteoglycans, which enable the normal function of the IVD.^9^ The progression of IDD is closely related to NP cells degeneration. During IDD, proinflammatory cytokines, such as TNF‐α and IL‐1β could accelerate NP cells degeneration by promoting the release of matrix metalloproteinases (MMPs) and inflammatory cytokines.^10^, ^11^, ^12^, ^13^ It has been confirmed that ECM degradation and inflammation play a critical role in accelerating IDD progression. However, the involved factors or mechanisms in the regulation of ECM metabolism and inflammation are not completely understood.

The nuclear factor‐kappa B (NF‐κB) signalling pathway has been demonstrated as a crucial mediator of the IDD process and the therapeutic target in disc degeneration.^12^, ^14^, ^15^, ^16^, ^17^ Pro‐inflammatory cytokines activate the NF‐κB signalling and downstream pathways, thereby participating in inflammatory response, cell apoptosis and ECM metabolism.^10^, ^17^ In the NF‐κB signalling pathway, TNF‐α binds to its receptor and transduces the signal to activate the IκB kinase (IKK) complex, which is composed of three units: IκB kinase α (IKKα), IκB kinase β (IKKβ) and IκB kinase γ (IKKγ). Activated IKK complex results in the phosphorylation of inhibitor of κB alpha (IκBα) and p65, which alter the downstream gene expression in NP cells.^17^, ^18^, ^19^ Hyperactivation of NF‐κB signalling is an essential step in promoting ECM catabolism and inflammatory cytokines production, which in turn could accelerate the progression of IDD.^12^

Accumulating evidence has showed that angiopoietin‐like proteins (ANGPTL) are associated with inflammation, metabolism, cell apoptosis and cancer progression.^20^, ^21^ ANGPTL proteins have eight family members and mainly are secreted glycoproteins with a structure similar to that of angiopoietin family proteins. Our previous study has indicated the role of angiopoietin family proteins in IDD.^22^ One of the members in ANGPTL family, angiopoietin‐like proteins 8 (ANGPTL8), which is also known as lipasin or betatrophin, is a regulator of plasma lipid metabolism and a therapeutic target in diabetes mellitus.^23^, ^24^, ^25^, ^26^, ^27^ Nevertheless, the role of ANGPTL8 in inflammation and related signalling pathway is unknown. A recent study has shown that ANGPTL8 has an intracellular location and was suggested to be related to the regulation of inflammation.^28^ Our work is the first study to investigate the role of ANGPTL8 in ECM metabolism and inflammation during the IDD process, and may provide a new potential target for the development of therapeutic strategies for IDD.

So far, the effect of ANGPTL8 on NP cells, as well as its role in inflammation and ECM degradation, remains unclear. Thus, to investigate the role of ANGPTL8 in IDD in this study, we first measured the level of ANGPTL8 expression in normal and degenerative human NP tissue samples. Subsequently, we investigated the effects of ANGPTL8 silencing or over‐expression on ECM degradation and inflammation in human NP cells, and elucidated the relationship between ANGPTL8 and the NF‐κB signalling pathway. Meanwhile, a rat degenerative disc model of ANGPTL8‐silencing was developed to clarify the effects in vivo.

## 2 MATERIALS AND METHODS

### 2.1 Collection of human NP tissues

The experimental protocols in this study were approved by the Ethics Committee of Tongji Medical College, Huazhong University of Science and Technology. Written informed consent was obtained from all participants included in our study. Human NP tissues were collected from 40 patients (men 16 and women 24; mean age 32.5 years, range 17‐56 years) who underwent surgery for idiopathic scoliosis or degenerative disc disease. The Pfirrmann grades based on T2‐weighted section images were used to assess the degree of IDD.^29^ In brief, Grade II showed an inhomogeneous disc with or without horizontal bands and a hyperintense white signal; Grade III showed an inhomogeneous disc with intermediate gray signal intensity and a slightly decreased disc height; Grade IV showed an inhomogeneous disc with hypointense gray or black signal intensity and a moderately decreased disc height; Grade V showed an inhomogeneous disc with a hypointense black signal intensity and a collapsed disc space. The specimens were immediately sectioned for use in various experiments. One section fixed in 4% buffered formaldehyde (pH 7.4) was used in histological analysis. A second section was immediately immersed in RNAlater (Invitrogen, Carlsbad, CA) and frozen in liquid nitrogen for protein and RNA analysis. A third section was covered with phosphate‐buffered saline (PBS) in a sterile tube for cell isolation.

### 2.2 Isolation and culture of NP cells

Nucleus pulposus cells were isolated as previously described.^22^ Briefly, NP tissues were cut into pieces and enzymatically digested in 0.2% type II collagenase and 0.25% trypsin for 3 hours. After filtering and washing with PBS, the suspension was centrifuged, and the isolated cells were cultured in Dulbecco's modified Eagle medium containing 15% foetal bovine serum (Gibco, Waltham, MA) and 1% penicillin/streptomycin (Invitrogen) in a 5% CO_2_ incubator. The culture medium was replaced twice a week, and the cells from the second or third passage were prepared for the subsequent experiments.

### 2.3 Quantitative real‐time polymerase chain reaction

Total RNA, extracted with Trizol reagent (Invitrogen) from NP tissues or cultured NP cells, was reverse‐transcribed and amplified by Quantitative real‐time polymerase chain reaction (qRT‐PCR) according to the standard protocols. The qRT‐PCR was performed to quantify ANGPTL8, COL2A1, MMP3, MMP9, IL‐6 and GAPDH mRNA expression levels. GAPDH was used for normalization. The primers used for qRT‐PCR are listed in Table 1. All the data were tested in triplicate.

**Table 1:** Sequences of primers used for quantitative real‐time PCR

Gene	Forward	Reverse	Size (bp)
ANGPTL8	GCCGCACAATAGAACTCCTG	CAAATTCTCGGTAGGCAGGG	237
COL2A1	AGAACTGGTGGAGCAGCAAGA	AGCAGGCGTAGGAAGGTCAT	142
MMP3	TTCCTTGGATTGGAGGTGAC	AGCCTGGAGAATGTGAGTGG	248
MMP9	GAGACCGGTGAGCTGGATAG	TACACGCGAGTGAAGGTGAG	236
IL‐6	TTCGGTCCAGTTGCCTTCTC	GCCTCTTTGCTGCTTTCACA	227
GAPDH	TCAAGAAGGTGGTGAAGCAGG	TCAAAGGTGGAGGAGTGGGT	115

### 2.4 Western blot analysis

Cell proteins were isolated using a protein extraction kit (Beyotime, Nantong, China) according to manufacturer instructions and analysis was performed using standard procedures. The following antibodies were used: ANGPTL8 (1:200), COL2A1 (1:5000), MMP3 (1:1000), MMP9 (1:800), IL‐6 (1:1000) and GAPDH (1:1000) (Abcam, Cambridge, MA). NF‐κB (p65) (1:2000), phosphorylated NF‐κB (p‐p65) (1:1000), IκBα (1:1000), phosphorylated inhibitor of κB alpha (p‐IκBα; 1:10000), IKKα (1:10000), IKKβ (1:800) and IKKγ (1:800) (Cell Signaling Technology, Danvers, MA). Horseradish peroxidase (HRP)‐conjugated secondary antibodies (Santa Cruz Biotechnology, Dallas, TX) were also used, and protein bands were visualized and detected using the enhanced chemiluminescence system. GAPDH were used as loading controls. The experiment was performed in triplicate.

### 2.5 Immunofluorescence analysis

Immunofluorescence analysis was performed as previously described.^30^ Briefly, NP cells or tissues attached to slides were fixed with 4% paraformaldehyde, and then permeabilized with 0.2% Triton X‐100 in PBS. The slides were washed in PBS and blocked with 2% bovine serum albumin (BSA) in PBS for 2 hours at 37°C, and then incubated with primary antibodies against: ANGPTL8 (1:150), COL2A1 (1:100), MMP3 (1:200), MMP9 (1:200) and IL‐6 (1:150). Nuclei were co‐stained for 5 minutes with 0.1 g/mL 4, 6‐diamidino‐2‐phenylindole (Beyotime, Nantong, China). Images were captured under a fluorescence microscope (Olympus, BX53; Melville, NY).

### 2.6 Enzyme‐linked immunosorbent assay

After the NP cells culture, supernatants were collected and centrifuged, the contents of ANGPTL8, MMP3 and MMP9 secreted by NP cells were analysed using the corresponding Enzyme‐linked immunosorbent assay (ELISA) kit (R&D Systems, Inc, Minneapolis, MN) according to the standard protocol. The experiment was performed in triplicate.

### 2.7 Knockdown or over‐expression of ANGPTL8

Nucleus pulposus cells were seeded and cultured in six‐well plates and reached 70%‐80% confluence before infection. Knockdown of ANGPTL8 in NP cells was realized by transfection with short interfering RNA (siRNA): siRNA‐231 sequence 5'‐GCCGCACAAUAGAACUCCUTT‐3', siRNA‐325 sequence 5'‐GGAUAUUCUGCAGCUGCAGTT‐3', siRNA‐457 sequence 5'‐CCGAGAAUUUGAGGUCUUATT‐3' and the scrambled siRNA sequence 5'‐UUCUCCGAACGUGUCACGUTT‐3'. SiRNA against ANGPTL8 (si‐ANGPTL8) and scrambled siRNA (si‐NC) were synthesized by RiboBio (Guangzhou, China), and cells were transfected with Lipofectamine 2000 (Invitrogen) according to the standard protocols. Moreover, to realize ANGPTL8 over‐expression, NP cells were infected with pLVX‐IRES‐ZsGreen1‐homo‐ANGPTL8 (over‐ANGPTL8) or pLVX‐IRES‐ZsGreen1 (over‐NC) according to the standard protocols. Transgenic expression in NP cells was detected using qRT‐PCR at 48 hours after transfection. Transfection efficiency was quantified by assessing ZsGreen‐positive cells under a fluorescence microscope (Olympus, BX53; Melville, NY).

### 2.8 Animal experiments

The animal experiments were performed following a protocol approved by the Animal Experimentation Committee of Huazhong University of Science and Technology. In total, 60 3‐month‐old Sprague‐Dawley rats purchased from Experimental Animal Center of Tongji Medical College, Huazhong University of Science and Technology were used for the in vivo experiments. A model of IDD was established by needle puncture. Except for 15 rats, which were used for negative control, all remaining animals underwent surgery by needle puncture. After the rats were anaesthetized with 2% (w/v) pentobarbital (40 mg/kg), the IVD of the rats (Co 8/9) was punctured by a 20‐gauge needle from the dorsal side.^31^ The second puncture was performed 1 week later, and the rats were randomly divided into three groups: non‐injection (IDD) group, group with injection of normal PBS (2 μL), and group with injection of ANGPTL8‐siRNA plasmid (2 μL, 20 μmol/L) using a 33‐gauge needle (Hamilton, Benade, Switzerland).^32^, ^33^ The injection procedure was repeated after 2 weeks.

### 2.9 Radiography and magnetic resonance imaging examination

All rats underwent radiography (X‐ray), and the disc height was measured using the ImageJ software (US National Institutes of Health) and calculated using the disc height index (DHI) according to a previously described method.^31^ Changes in the DHI of the punctured IVDs were defined as %DHI (%DHI = post‐punctured DHI/pre‐punctured DHI*100%).^31^, ^34^ Moreover, magnetic resonance imaging (MRI) was performed using a 7.0 T animal specific MRI system (BRUKER BioSpec 70/20 USR, Germany) and sagittal T2‐weighted images were captured to evaluate the signal changes of the discs. T2‐weighted sections in the sagittal plane were obtained using the following parameters: a fast‐spin echo sequence with a time‐to‐repetition of 2000 ms and a time‐to‐echo of 36 ms, a 256 (h) ×256 (v) matrix, a field of view 6.00/3.00 cm, a flip angle of 180°. Pfirrmann grades according to T2‐weighted section images were used to assess the degree of IDD, as previously described.^34^

### 2.10 Histologic analysis

Animals were euthanized and the discs were harvested. The specimens were decalcified and fixed in formaldehyde, dehydrated and embedded in paraffin. The slides of each disc were stained with haematoxylin and eosin (HE) staining and assessed using a histological grading scale.^34^ The specimen sections were deparaffinized and rehydrated, and then microwaved in 0.01 mol/L sodium citrate for 15 minutes. Next, 3% hydrogen peroxide was used to block endogenous peroxidase activity for 10 minutes, and 5% BSA was used to block non‐specific binding sites for 30 minutes. The sections were then incubated with the primary antibodies (MMP3, 1:200) and (COL2A1, 1:100) overnight at 4°C. Finally, the sections were incubated with an HRP‐conjugated secondary antibody and counterstained with haematoxylin. All tests were performed with at least three sections from each specimen.

### 2.11 Statistical analysis

All experiments were performed independently at least in triplicate and data are presented as the mean ± SD. Statistical analyses were performed using GraphPad Prism 7 software (La Jolla, CA). Differences between group means were evaluated using Student's *t* test or one‐way ANOVA. *P* < 0.05 was considered statistically significant.

## 3 RESULTS

### 3.1 Expression of ANGPTL8 in human NP tissues during degeneration

**Figure 1:**
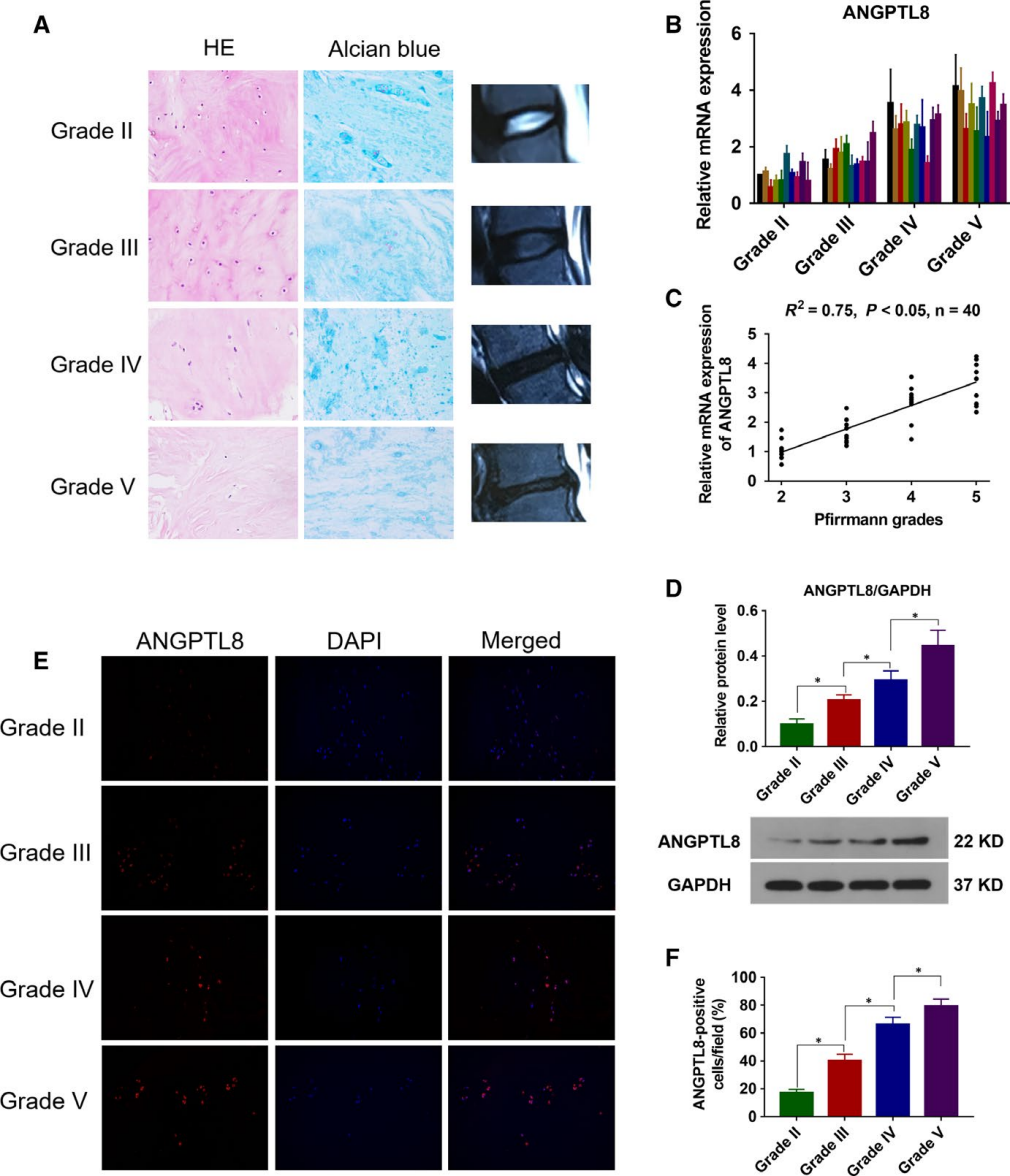
Angiopoietin‐like protein 8 (ANGPTL8) expression in human NP tissues. (A), Representative histological images of different Pfirrmann degrees NP tissues in HE and Alcian blue staining. Magnification: 400×. (B), ANGPTL8 mRNA level was measured by qRT‐PCR in different Pfirrmann grades of NP tissues. (C), Correlation between ANGPTL8 mRNA level and Pfirrmann grades of NP tissues (n = 40) analysed by non‐parametric linear regression. (D), Representative images and the quantitative statistical analysis of ANGPTL8 protein expression according to the Western blot analysis. GAPDH was used as an internal control. (E,F), Representative images of ANGPTL8 expression and statistical analysis of positive ANGPTL8 cells as detected by immunofluorescence analysis. Magnification: 200×. Data were presented as the mean ± SD (n = 3). *P < 0.05

The degree of IDD was assessed by MRI in T2‐weighted images, and 10 specimens of each degenerative degree were selected in the present study. HE staining and Alcian blue staining were used to confirm the degenerative degree of selected IVD tissues (Figure 1A). To investigate the expression of ANGPTL8 in the process of the IDD, the transcriptional level was measured by qRT‐PCR in samples with different IDD degrees (Figure 1B). A positive correlation was found between ANGPTL8 mRNA level and IDD grade (Figure 1C). Moreover, Western blot analysis indicated that a higher ANGPTL8 protein expression level was related to a higher degenerative grade of NP tissues (Figure 1D). Additionally, the positive rate of ANGPTL8‐labelled cells was significantly greater in Grade IV or V than in Grade II or III according to the immunofluorescence analysis (Figure 1E‐F). These results demonstrated that the level of ANGPTL8 expression in human NP tissues was increased during the IDD process.

### 3.2 TNF‐α treatment facilitate the expression of ANGPTL8 in NP cells

**Figure 2:**
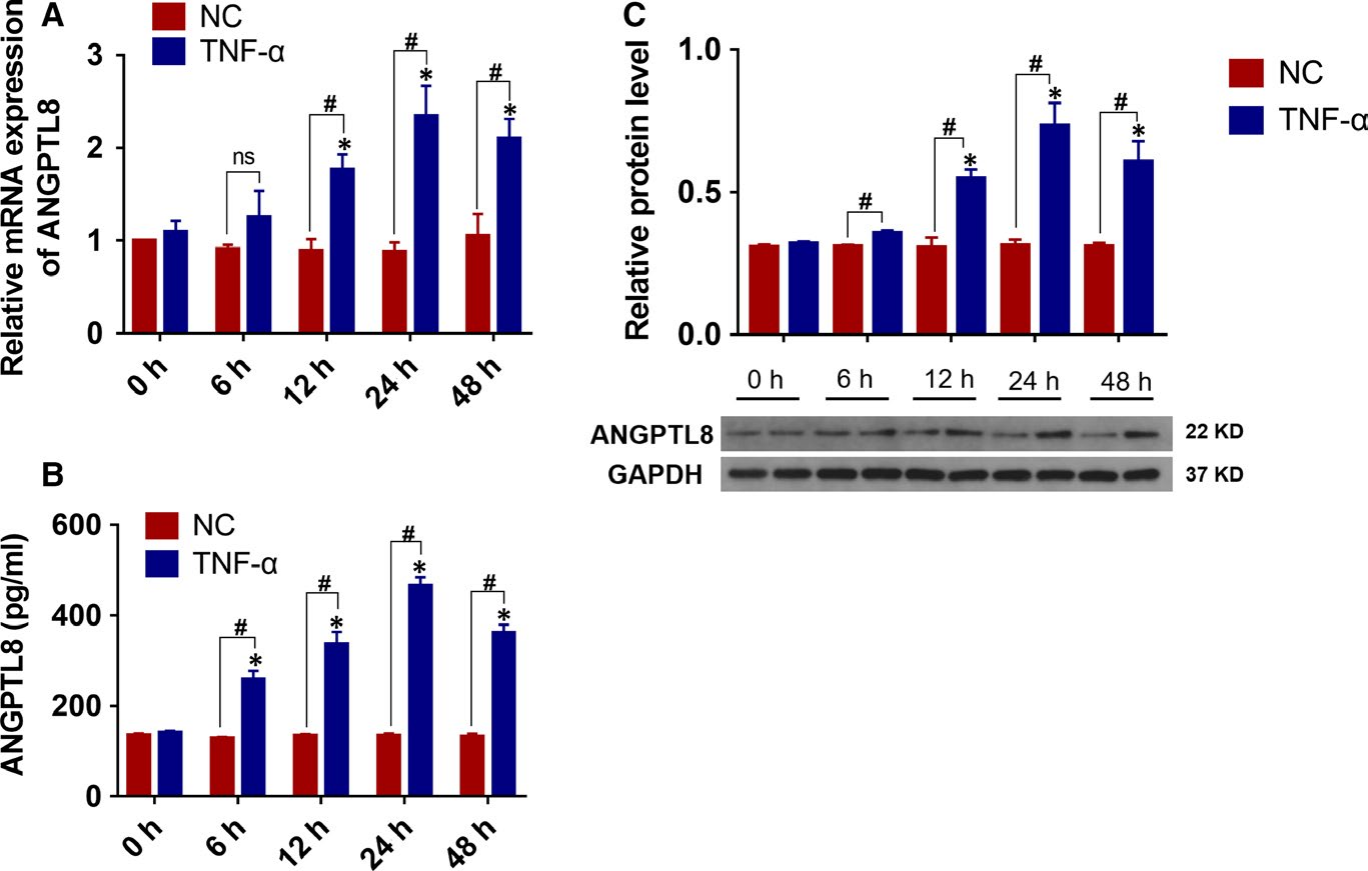
TNF‐α promoted the expression of Angiopoietin‐like protein 8 (ANGPTL8) in human NP cells. (A), The human NP cells were treated with TNF‐α (50 ng/mL) in 0, 6, 12, 24, 48h, and the mRNA level of ANGPTL8 was analysed by qRT‐PCR. (B), Representative images and the quantitative statistical analysis of ANGPTL8 protein expression according to the Western blot analysis. GAPDH was used as an internal control. (C), The content of ANGPTL8 in culture medium supernatant was also measured by ELISA at different time points. Data were presented as the mean ± SD (n = 3). *P < 0.05 vs NC 0 h group, ^#^ *P* < 0.05 vs corresponding NC group

Previous study has demonstrated that pro‐inflammatory cytokines promoted the NP cells degeneration and accelerated the progression of IDD.^18^, ^35^ In order to investigate the pathological effect of ANGPTL8 expression in IDD, NP cells were treated with TNF‐α (50 ng/mL) for 0, 6, 12, 24 or 48 hours in vitro. In a time‐dependent manner, TNF‐α treatment up‐regulated the transcriptional level of ANGPTL8 significantly (Figure 2A). Similarly, the protein level of ANGPTL8 was also increased due to the stimulation of TNF‐α (Figure 2B). Moreover, the contents of ANGPTL8 in NP cells culture supernatant were increased in a time‐dependent manner as analysed by ELISA (Figure 2C). Overall, these findings indicated that TNF‐α treatment could up‐regulate the level of ANGPTL8 expression in NP cells.

### 3.3 Knockdown of ANGPTL8 reduces TNF‐α‐induced ECM degradation and inflammation in vitro

**Figure 3:**
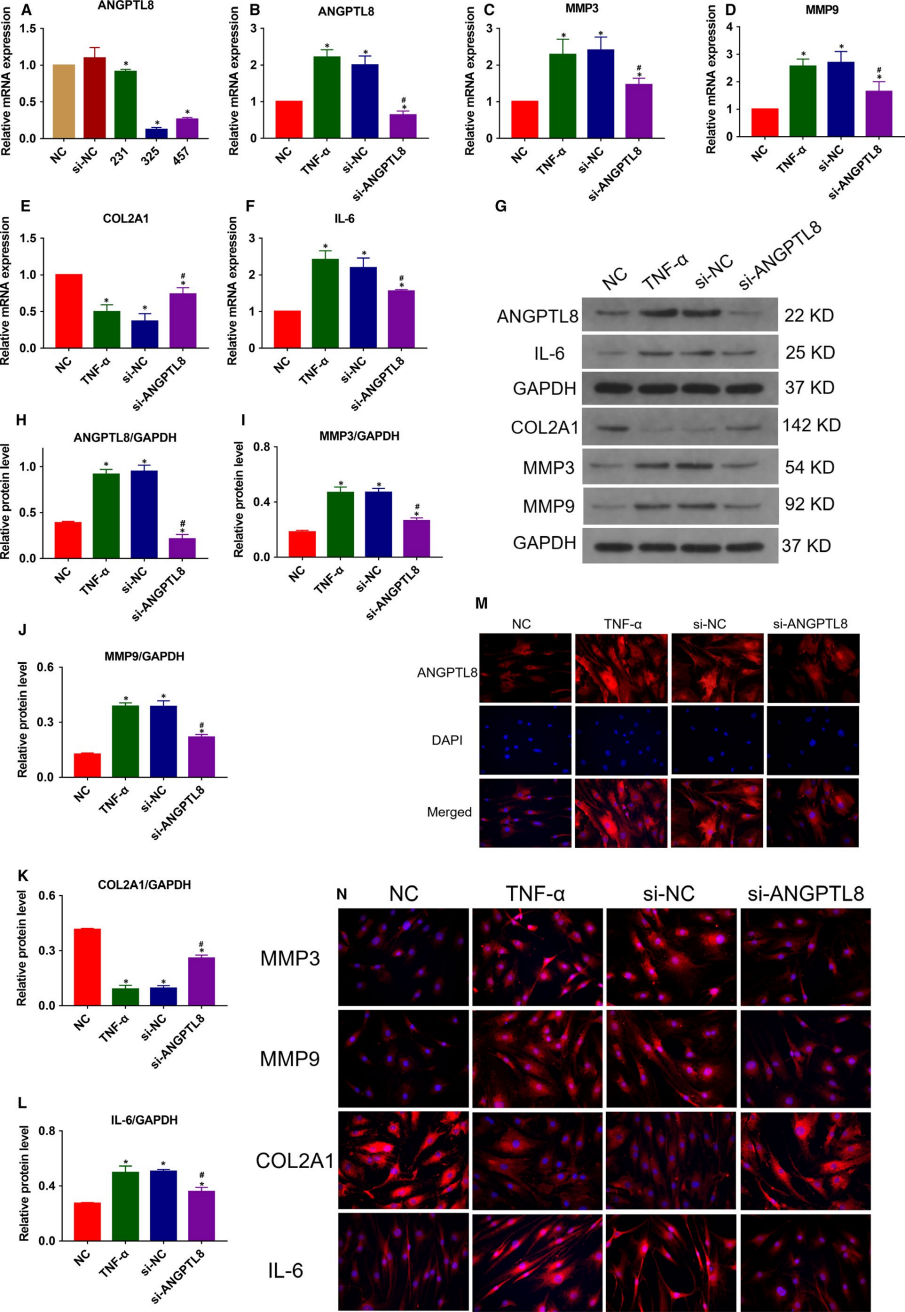
Knockdown of Angiopoietin‐like protein 8 (ANGPTL8) attenuated TNF‐α‐induced extracellular matrix (ECM) degradation and inflammation in human NP cells. (A), The human NP cells were transfected with si‐ANGPTL8 or the scrambled control (si‐NC), and the inhibitory efficiency was tested by qRT‐PCR. (B‐F) The human NP cells were treated with TNF‐α (50 ng/mL) except for the NC group. The mRNA expression level of ANGPTL8 (B), MMP3 (C), MMP9 (D), COL2A1 (E), IL‐6 (F) were measured after the NP cells were treated with siRNA. (G‐L), Western blot analysis and the quantitative statistical analysis showed the protein expression level of ANGPTL8 (H), MMP3 (I), MMP9 (J), COL2A1 (K), IL‐6 (L) in diffferent groups. GAPDH was used as an internal control. Data were presented as the mean ± SD (n = 3). *P < 0.05 vs NC group, ^#^ *P* < 0.05 vs TNF‐α group. (M), Representative images of ANGPTL8 expression were showed by immunofluorescence analysis. Magnification: 400×. (N), Representative images of MMP3, MMP9, COL2A1 and IL‐6 protein expression were detected by immunofluorescence staining. Magnification: 400×

To determine whether ANGPTL8 plays a role in TNF‐α induced inflammation and ECM degradation in NP cells, knockdown of ANGPTL8 was realized by transfection with siRNA (si‐ANGPTL8). Three si‐ANGPTL8 fragments were generated, and the inhibitory efficiency of ANGPTL8 in NP cells was analysed (Figure 3A). The si‐ANGPTL8 fragments with the most effective inhibitory efficiency (si‐325) were used for transfection. The NP cells were transfected with si‐ANGPTL8 under the stimulation of TNF‐α. The transcriptional and protein levels of type II collagen, MMP3, MMP9 and IL‐6 were measured (Figure 3B‐L). As expected, TNF‐α treatment induced the catabolism of ECM, which was characterized by decreased type II collagen expression, increased MMP3 and MMP9 expression, and the production of inflammatory cytokine, IL‐6. Interestingly, inhibition of ANGPTL8 resulted in the repression of MMP3, MMP9 and IL‐6 expression and the up‐regulation of type II collagen expression. Moreover, these variation profiles in immunofluorescence analysis were consistent with the results of Western blot analysis. The fluorescence intensity of MMP3, MMP9 and IL‐6 were decreased whereas that of type II collagen increased in the si‐ANGPTL8 group compared with those in the control group (Figure 3M‐N). Hence, knockdown of ANGPTL8 attenuated the production of MMPs and inflammatory cytokines, and preserved type II collagen contents in NP cells.

### 3.4 Over‐expression of ANGPTL8 promotes TNF‐α‐induced ECM degradation and inflammation in vitro

**Figure 4:**
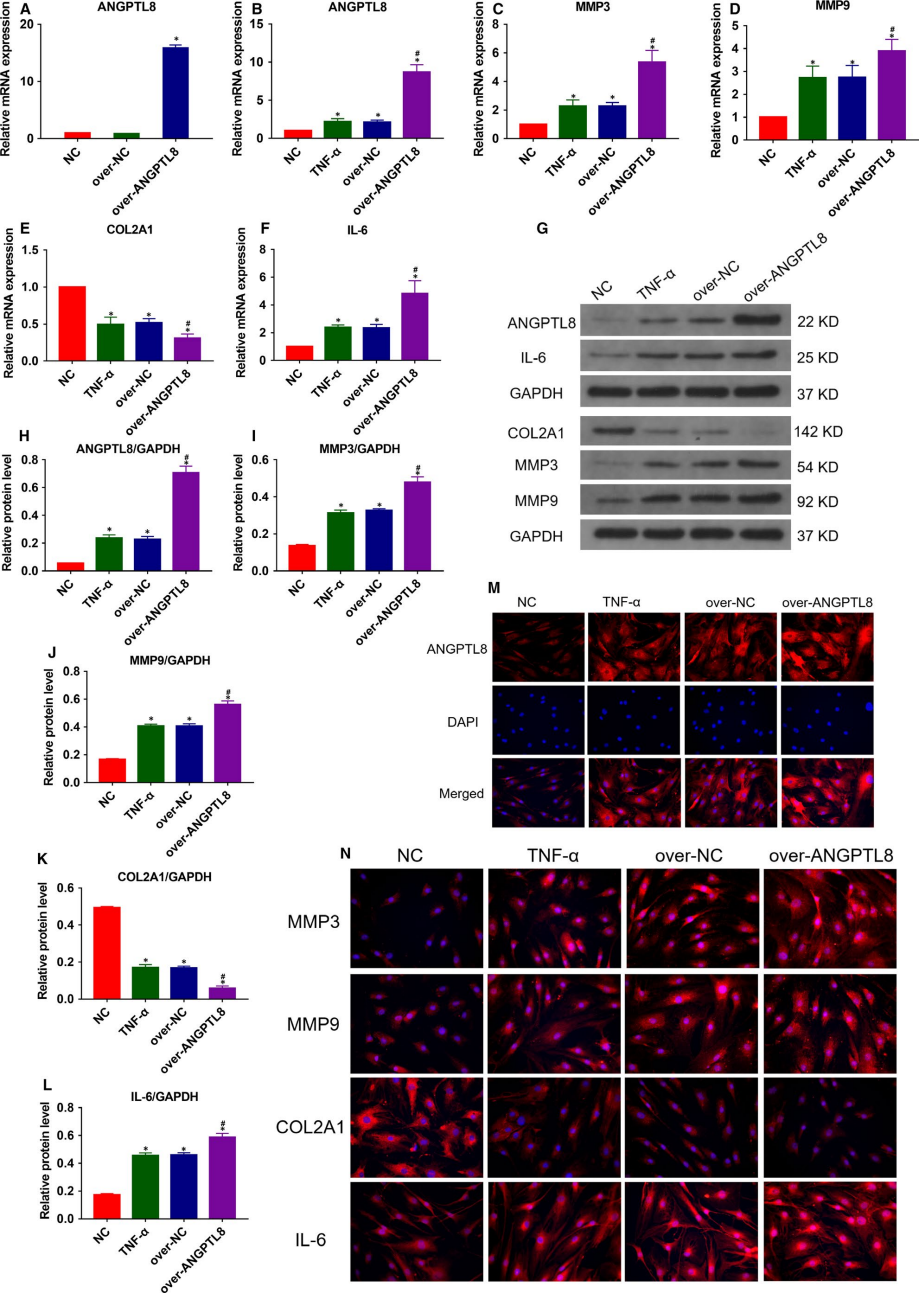
Overexpression of Angiopoietin‐like protein 8 (ANGPTL8) exacerbated TNF‐α‐induced extracellular matrix (ECM) degradation and inflammation in human NP cells. (A), The NP cells were transfected with ANGPTL8 lentivirus vector encoding or control vector and the expression efficiency was measured. (B) The human NP cells were treated with TNF‐α (50 ng/mL) except for the NC group. The mRNA expression level of ANGPTL8 (B), MMP3 (C), MMP9 (D), COL2A1 (E), IL‐6 (F) were analysed in the ANGPTL8‐overexpressed NP cells by qRT‐PCR. (G‐L) The protein expression level of ANGPTL8 (H), MMP3 (I), MMP9 (J), COL2A1 (K), IL‐6 (L) were measured by Western blot and analysed statistically in different groups. GAPDH was used as an internal control. Data were presented as the mean ± SD (n = 3). *P < 0.05 vs NC group, ^#^ *P* < 0.05 vs TNF‐α group. (M), Representative images of ANGPTL8 expression were showed by immunofluorescence analysis. Magnification: 400×. (N), Representative images of MMP3, MMP9, COL2A1 and IL‐6 protein expression were also detected by immunofluorescence staining. Magnification: 400×

In order to further assess the effect of ANGPTL8 during IDD, NP cells were transfected with lentivirus vector encoding homo‐ANGPTL8, and the transgenic efficiency was evaluated by qRT‐PCR (Figure 4A). The transcriptional and protein levels of type II collagen, MMP3, MMP9 and IL‐6 were analysed under the TNF‐α stimulation (Figure 4B‐L). Over‐expression of ANGPTL8 resulted in a significant up‐regulation of MMP3, MMP9 and IL‐6, and promoted the degradation of type II collagen compared with both the control group and TNF‐α group. Similarly, immunofluorescence analysis also indicated that an elevated level of ANGPTL8 reduced the deposition of type II collagen and increased the MMP3, MMP9 and IL‐6 protein level (Figure 4M‐N). These data demonstrated that ANGPTL8 enhanced the detrimental effects of TNF‐α during the NP cells degeneration by promoting the release of MMPs and inflammatory cytokines.

### 3.5 ANGPTL8 regulates ECM metabolism and inflammatory response through the NF‐κB signalling pathway

**Figure 5:**
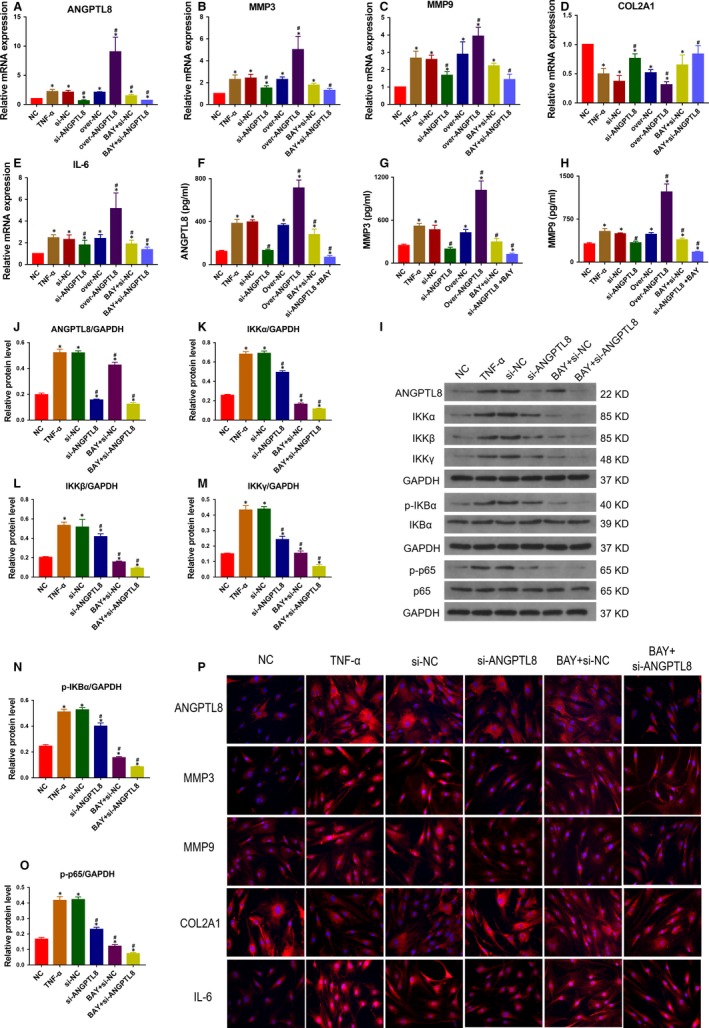
Angiopoietin‐like protein 8 (ANGPTL8) promoted extracellular matrix (ECM) degradation and inflammation through NF‐κB signalling pathway in human NP cells. The human NP cells were treated with TNF‐α (50 ng/mL) except for the NC group. (A‐E), The mRNA expression level of ANGPTL8 (A), MMP3 (B), MMP9 (C), COL2A1 (D), IL‐6 (E) were measured respectively by qRT‐PCR in ANGPTL8 silencing or overexpression treated with BAY‐11‐7082 or not. (F‐H), ELISA kits were used to detect the secreted ANGPTL8 (F), MMP3 (G), MMP9 (H) respectively in different groups. (I), The components of IKK complex and the downstream phosphorylation level of IκBα and p65 were analysed by Western blot analysis. (J‐O), The relative protein level of ANGPTL8 (J), IKKα (K), IKKβ (L), IKKγ (M), phosphorylated IκBα (N) and p65 (O) were analysed by the protein quantification. GAPDH was used as an internal control. Data were presented as the mean ± SD (n = 3). *P < 0.05 vs NC group, ^#^ *P* < 0.05 vs TNF‐α group. (P), Representative images of ANGPTL8, MMP3, MMP9, COL2A1 and IL‐6 protein expression were showed by immunofluorescence staining. Magnification: 400×

The over‐activation of the NF‐κB signalling pathway is associated with the pathogenesis of IDD.^36^ Pro‐inflammatory cytokines, such as TNF‐α, bind to specific receptors in the cytomembrane and activate the classical NF‐κB pathway.^37^ It was assumed that ANGPTL8 could exacerbate the adverse effects of TNF‐α through the regulation of NF‐κB signalling in NP cells. To test the hypothesis, the NF‐κB pathway inhibitor, BAY‐11‐7082 was treated with TNF‐α and the expression level of ANGPTL8, MMP3, MMP9, COL2A1 and IL‐6 were measured (Figure 5A‐E). Moreover, the expression profile in the culture supernatants of NP cells was consistent with the intracellular ANGPTL8, MMP3 and MMP9 in the ELISA analysis (Figure 5F‐H).

To verify this hypothesis further, the protein level of NF‐κB signalling downstream targets were measured (Figure 5I‐O). TNF‐α treatment activated the IKK complex, which resulted in increased IKKα, IKKβ and IKKγ levels. Activation of IKK complex resulted in the phosphorylation of IκBα, then promoting the phosphorylation and nucleus translocation of p65, thereby resulting in the downstream gene expression. Silencing of ANGPTL8 decreased the protein level of three members of the IKK complex. Phosphorylated IκBα and p65 were also decreased in the si‐ANGPTL8 group compared with those in the TNF‐α treatment group. Interestingly, si‐ANGPTL8 transfection achieved the inhibitory effects of NF‐κB signalling that are consistent with the effects of BAY‐11‐7082. Moreover, when treated with si‐ANGPTL8 and BAY‐11‐7082, the inactivation of NF‐κB signalling pathway was much more significant under TNF‐α stimulation in NP cells. Accordingly, the immunofluorescence staining results of ANGPTL8, MMP3, MMP9, COL2A1 and IL‐6 in different groups also suggested that ANGPTL8 was closely related to the activation of NF‐κB signalling and the expression of downstream genes (Figure 5P). These data suggested that ANGPTL8 promoted ECM degradation and inflammatory cytokine release through activating the NF‐κB signalling pathway, thereby indicating the detrimental role of ANGPTL8 during IDD.

### 3.6 Silencing of ANGPTL8 ameliorates IVD degeneration in vivo

**Figure 6:**
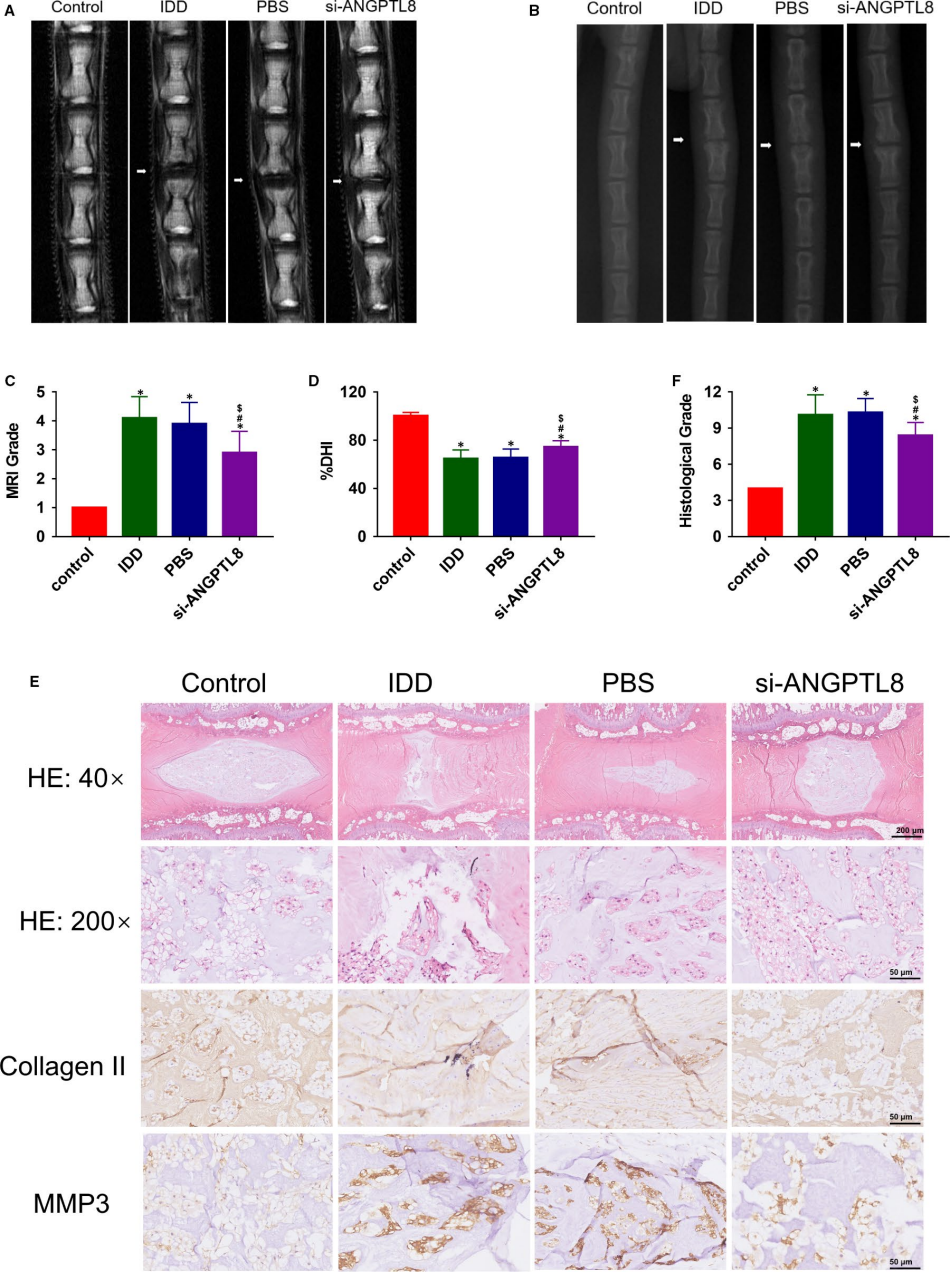
Silencing of Angiopoietin‐like protein 8 (ANGPTL8) attenuated the progression of IDD in a puncture‐induced disc degeneration model. (A), T2‐weighted magnetic resonance imaging (MRI) of rat tail with a needle‐punctured disc at 8 wk in each group (white arrows). (B), X‐ray of rat tail with a needle‐punctured disc at 8 wk in each group (white arrows). (C), Pfirrmann MRI grade scores on the basis of the T2‐weighted MRI results in each group. (D), Changes in DHI (%DHI) based on the X‐ray results in each group. (E), Representative HE staining and immunohistochemical staining of COL2A1, MMP3 expression in each group. Magnification: 40× (scar bar = 200 μm) and 200× (scar bar = 50 μm). (F), Histological grades were measured according to the HE staining results. Data were presented as the mean ± SD (n = 3). *P < 0.05 vs control group, ^#^ *P* < 0.05 vs IDD group. ^$^ *P* < 0.05 vs PBS group

To evaluate the effects of ANGPTL8 in vivo, intradiscal delivery of si‐ANGPTL8 was performed in a rat degeneration disc model. The extent of IDD was assessed by MRI and X‐ray examination at 8 weeks (Figure 6A,B). According to the MRI results, the Pfirrmann score, which was used to evaluate the degree of IDD, was lower in the si‐ANGPTL8 group than in the IDD or PBS group (Figure 6C). In addition, the %DHI was calculated based on the X‐ray results. A low %DHI indicated a degenerative disc with a collapsed or narrowing intervertebral space, and it was noted that silencing of ANGPTL8 could restore the disc height to some extent (Figure 6D). In order to further investigate the changes of IDD, histological staining and immunohistochemistry analysis were performed for the rat disc (Figure 6E). Each group was subjected to HE staining and was assessed by histological grading (Figure 6F). The si‐ANGPTL8 group showed a higher level of type II collagen and a lower level of MMP3, and also showed improved histological grade compared with the IDD or PBS group. These results that knockdown ANGPTL8 could significantly retard the progression of IDD.

## 4 DISCUSSION

**Figure 7:**
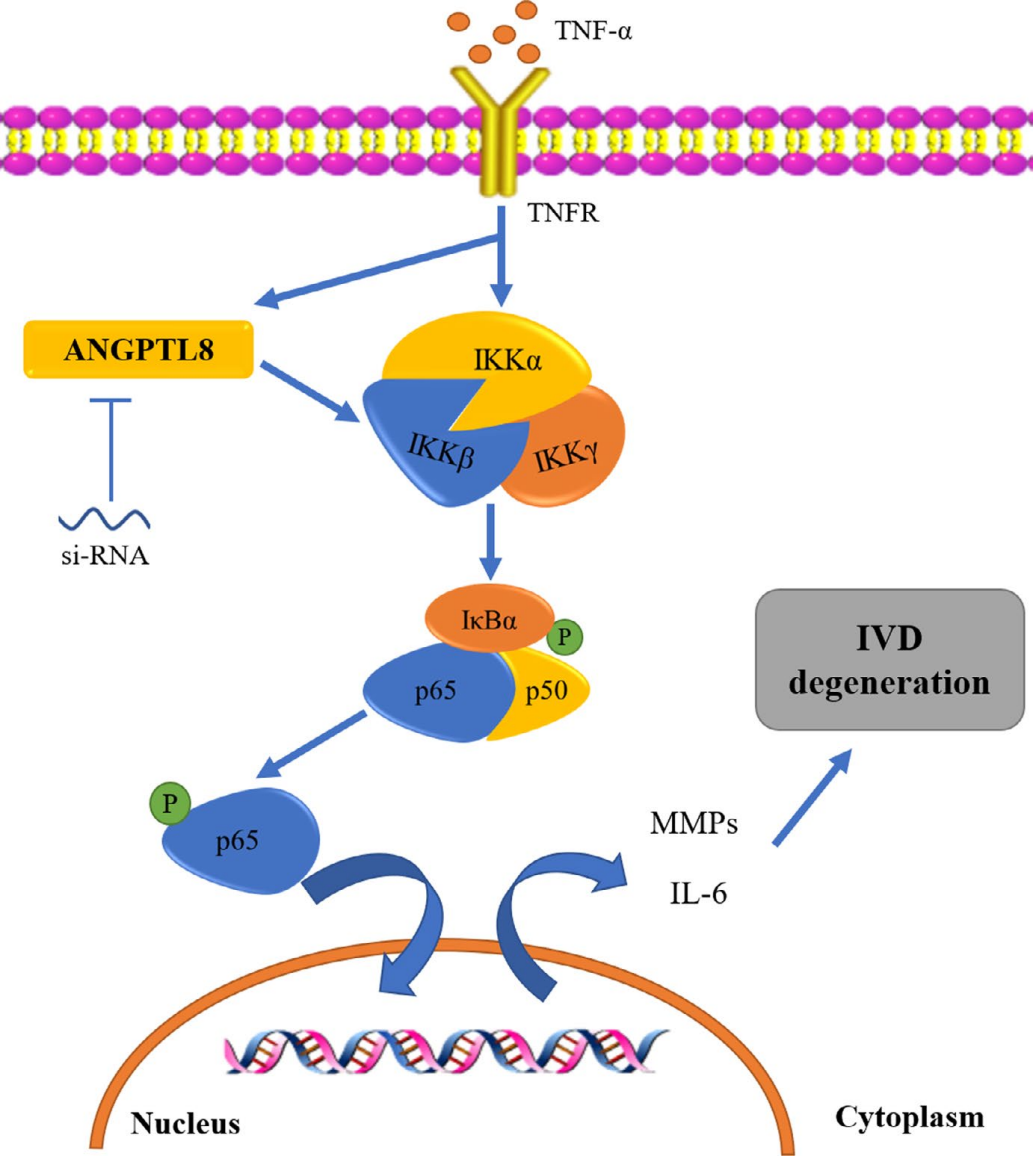
Schematic graph of the role of ANGPTL8 in TNF‐α‐induced human NP cells ECM catabolism and inflammation. The increased expression of ANGPTL8 was related to ECM degradation and inflammation through enhancing the NF‐κB signalling pathway in TNF‐α‐treated NP cells. Inhibiting of ANGPTL8 partly attenuated the activation of the NF‐κB signalling, and reduced the downstream MMPs and IL‐6 expression. ANGPTL8, angiopoietin‐like protein 8; TNF‐α, tumour necrosis factor α; TNFR, receptor of TNF‐α; NF‐κB, nuclear factor‐kappa B; IKKα, the IκB kinase α; IKKβ, the IκB kinase β; IKKγ, the IκB kinase γ; IκBα, inhibitor of NF‐κB α; IVD, intervertebral disc; NP, nucleus pulposus; ECM, extracellular matrix; MMPs, matrix metalloproteinases; IL‐6, interleukin‐6

Imbalance between ECM metabolism and inflammatory response in NP tissue of IVD is the critical culprit in the development of IDD.^30^ In the present study, we found that the expression level of ANGPTL8 was increased in degenerative disc and correlated with the degree of IDD. Knockdown of intracellular ANGPTL8 expression in NP cells reduced the expression of MMP3 and MMP9, and the production of the pro‐inflammatory cytokine, IL‐6. On the contrary, the content of type II collagen was increased. Accordingly, TNF‐α treatment promoted the expression of ANGPTL8 and over‐expression of ANGPTL8 under TNF‐α stimulation showed the opposite effects to that with ANGPTL8 silencing. Our further study indicated that ANGPTL8 promoted the TNF‐α‐induced ECM degradation and inflammation through the NF‐kB signalling pathway in NP cells (Figure 7). In addition, the in vivo study in a rat tail puncture‐induced degeneration model also demonstrated that silencing ANGPTL8 could partly restore the degenerative IVD. These findings indicated that ANGPTL8 plays an adverse role during the IDD process.

The ECM of the NP tissue is rich in collagens and proteoglycans.^17^ Approximately 70% of NP dry weight, which is involved in water absorption, nutrition diffusion and osmotic regulation in NP tissues, is composed of type II collagen and aggrecan.^38^ In a healthy IVD, there is a harmonious balance between the catabolism and anabolism of ECM gradients, which is crucial for normal structure and function of IVD.^39^, ^40^ During the IDD process, the imbalance of ECM metabolism, presented as the progressive loss of type II collagen and aggrecan, induces changes of the morphology and structure of IVD, including disc height reduction and collapsed disc space, or decreased water content and mechanical buffering ability.^38^, ^41^, ^42^, ^43^, ^44^ In molecular analysis, catabolic enzymes, primarily MMPs and a disintegrin and metalloprotease with thrombospondin motifs (ADAMTSs), are highly expressed in degenerative IVD.^45^, ^46^ MMPs, such as MMP‐3, MMP‐9 and MMP‐13, degrade the type II collagen and aggrecan in ECM, resulting in the hyperactive catabolic effect.^38^, ^47^ Previous studies indicated that proinflammatory cytokines could promote the expression of MMPs and ADAMTSs, and accelerate the progression of IDD.^16^, ^48^, ^49^, ^50^ In the present experiment, TNF‐α treatment induced the expression of MMP3, MMP9 and IL‐6, as well as the decrease of type II collagen. It was demonstrated that TNF‐α accelerated the NP cells degeneration, and ANGPTL8 may serve as a pro‐degeneration molecule by enhancing the effects of pro‐inflammatory cytokines.

The NF‐κB signalling pathway has been verified to play a role in promoting the apoptosis and senescence of NP cells, and increasing matrix‐degrading enzyme activity and mediating inflammatory response in IDD.^16^, ^51^ Thus, inhibiting the NF‐κB signalling pathway has a therapeutic potential in the progression of IDD.^12^, ^14^, ^17^, ^51^, ^52^ In the NF‐κB signalling pathway, activation of IKK complex results in the phosphorylation of IκBα and p65. The p65 phosphorylation activates the downstream gene expression and cytokines release, such as that of catabolic genes (MMP3, MMP9, MMP13, ADAMTSs) and pro‐inflammatory factors.^14^ Our study demonstrated that knockdown of ANGPTL8 attenuated the activation of the NF‐κB signalling pathway, which in turn decreased the level of the IKK complex and promoted the dephosphorylation of IκBα and p65. Moreover, over‐expression of ANGPTL8 could enhance the effects of NF‐κB signalling. BAY‐11‐7082, which is a typical inhibitor of the NF‐κB, has the same effect as that of silencing of ANGPTL8 in NP cells under TNF‐α stimulation.^53^ The treatment of BAY‐11‐7082 also enhanced the effect of si‐ANGPTL8 on suppressing the NF‐κB activation. In all, these results supported the fact that ANGPTL8 promoted the activation of NF‐κB signalling and may be related to the hyperactive NF‐κB signalling during the NP cells degeneration.

Further, we evaluated the role of ANGPTL8 in a rat model of IDD in vivo. Needle puncture induced the development of IDD, which has been verified in various animals and clinical studies.^34^, ^54^, ^55^ Needles with a large diameter (20‐gauge) were used to induce the degeneration of the disc, while needles with a smaller diameter (33‐gauge) which could not exacerbate IDD were selected for the solution injection.^32^ The degree of IDD was assessed based on the histological changes or the morphology, water content, and disc height of IVD. Consistent with the in vitro study results, delivering si‐ANGPTL8 plasmids ameliorated the disc degeneration in vivo, which was presented as an improved histological grade in HE staining. The collagen II expression was increased and MMPs were decreased in the si‐ANGPTL8‐treated group compared with the IDD or PBS group. In addition, the conclusion that silencing of ANGPTL8 reversed the IDD process could also be supported by the MRI or X‐ray assessment and histological results. Thereby, our animal study suggested that silencing of ANGPTL8 slowed the progression of IDD to some degree.

Members of angiopoietin‐like proteins are associated with inflammation and metabolism, and most of them are pro‐inflammatory molecules.^20^ For example, ANGPTL2 and ANGPTL4 are acknowledged for their adverse pro‐inflammatory properties, and play a crucial role in mediating acute inflammation.^56^, ^57^ ANGPTL proteins could bind to specific receptors to activate the NF‐κB signalling pathway thereby resulting in the expression of inflammatory cytokines.^57^ However, the effect of ANGPTL8 on ECM metabolism and inflammation still remains unknown. A recent study showed that ANGPTL8 was down‐regulated in adipocytes due to the inflammation regulation; however, the mechanisms of ANGPTL8 in regulating inflammation have not been investigated.^58^ In our study, a higher level of ANGPTL8 expression was detected in the more degenerative NP tissues and ANGPTL8 was up‐regulated in NP cells under TNF‐α stimulation. ANGPTL8 enhanced the effects of TNF‐α in promoting ECM degradation and inflammation by hyperactivation of the NF‐κB signalling pathway. Moreover, the role of ANGPTL8 as a pro‐inflammatory molecule in the IDD was also confirmed in the animal experiments. However, our results seem to contradict with the results of Zhang et al^28^ They found that ANGPTL8 inhibited the activation of NF‐κB and downstream NF‐κB signalling in LPS‐induced inflammation model. The opposite results may be explained as follows: first, the NP cells used in our study are different from the cells in their study, and ANGPTL proteins may function quite diversely in different cells types. Second, it was confirmed that chronic and not acute inflammation promotes the IDD progression, and an acute inflammation model in their study may have a tremendous difference with IDD model.^59^, ^60^ Our study revealed that ANGPTL8 participates in the regulation of ECM metabolism and inflammation partly through the NF‐κB signalling pathway; nevertheless, the more involved mechanisms are still unknown. Thus, further studies are needed to fully evaluate the role of ANGPTL8 in the progression of IDD.

Although our study demonstrated the pro‐degeneration role of ANGPTL8 in NP cells, there still are several limitations in our study. First, besides the nucleus pulposus, the progression of IDD also is associated with the degeneration of endplate cartilage or annulus fibrosus. Thus, further studies involving other types of cells that are involved in the IDD process should be conducted. Second, we only investigated the role of intracellular ANGPTL8 by over‐expression or silencing during NP cells degeneration. Extracellular ANGPTL8 and the unknown binding receptor may also be involved in IDD. Thus, this should be investigated in the future study as well. Third, regardless of the fact that ANGPTL8 influences ECM degradation and inflammation through NF‐κB signalling, whether other signalling pathways or regulators are involved in IDD process remains unclear. Hence, further research and clinical studies on the role of ANGPTL8 in IDD process are needed.

In conclusion, our results indicated that ANGPTL8 plays an adverse role in the progression of IDD. Silencing of ANGPTL8 could reduce ECM degradation and inflammation in human NP cells via inhibition of the NF‐κB signalling pathway. Moreover, over‐expression of ANGPTL8 enhanced the effects of TNF‐α and the activation of NF‐κB signalling pathway. We also demonstrated that knockdown of ANGPTL8 ameliorates IDD progression in a rat model of IDD. Thus, ANGPTL8 may be a potential therapeutic target for the treatment of IDD.

## CONFLICTS OF INTEREST

All authors declare that there are no conflicts of interest.

## References

[1] Vergroesen PP , Kingma I , Emanuel KS , et al. Mechanics and biology in intervertebral disc degeneration: a vicious circle. Osteoarthritis Cartilage. 2015;23:1057‐1070. 25827971 10.1016/j.joca.2015.03.028

[2] Teraguchi M , Yoshimura N , Hashizume H , et al. Progression, incidence, and risk factors for intervertebral disc degeneration in a longitudinal population‐based cohort: the Wakayama Spine study. Osteoarthritis Cartilage. 2017;25:1122‐1131. 28089899 10.1016/j.joca.2017.01.001

[3] Dario AB , Ferreira ML , Refshauge KM , Lima TS , Ordonana JR , Ferreira PH . The relationship between obesity, low back pain, and lumbar disc degeneration when genetics and the environment are considered: a systematic review of twin studies. Spine J. 2015;15:1106‐1117. 25661432 10.1016/j.spinee.2015.02.001

[4] Sivan SS , Wachtel E , Roughley P . Structure, function, aging and turnover of aggrecan in the intervertebral disc. Biochem Biophys Acta. 2014;1840:3181‐3189. 25065289 10.1016/j.bbagen.2014.07.013

[5] Teraguchi M , Yoshimura N , Hashizume H , et al. Prevalence and distribution of intervertebral disc degeneration over the entire spine in a population‐based cohort: the Wakayama Spine Study. Osteoarthritis Cartilage. 2014;22:104‐110. 24239943 10.1016/j.joca.2013.10.019

[6] Sakai D , Grad S . Advancing the cellular and molecular therapy for intervertebral disc disease. Adv Drug Deliv Rev. 2015;84:159‐171. 24993611 10.1016/j.addr.2014.06.009

[7] Priyadarshani P , Li Y , Yao L . Advances in biological therapy for nucleus pulposus regeneration. Osteoarthritis Cartilage. 2016;24:206‐212. 26342641 10.1016/j.joca.2015.08.014

[8] Huang YC , Urban JP , Luk KD . Intervertebral disc regeneration: do nutrients lead the way? Nat Rev Rheumatol. 2014;10:561‐566. 24914695 10.1038/nrrheum.2014.91

[9] Wang F , Cai F , Shi R , Wang XH , Wu XT . Aging and age related stresses: a senescence mechanism of intervertebral disc degeneration. Osteoarthritis Cartilage. 2016;24:398‐408. 26455958 10.1016/j.joca.2015.09.019

[10] Wang X , Wang H , Yang H , et al. Tumor necrosis factor‐alpha‐ and interleukin‐1beta‐dependent matrix metalloproteinase‐3 expression in nucleus pulposus cells requires cooperative signaling via syndecan 4 and mitogen‐activated protein kinase‐NF‐κB axis: implications in inflammatory disc disease. Am J Pathol. 2014;184:2560‐2572. 25063530 10.1016/j.ajpath.2014.06.006 PMC4188173

[11] Risbud MV , Shapiro IM . Role of cytokines in intervertebral disc degeneration: pain and disc content. Nat Rev Rheumatol. 2014;10:44‐56. 24166242 10.1038/nrrheum.2013.160 PMC4151534

[12] Tu J , Li W , Zhang Y , et al. Simvastatin inhibits IL‐1beta‐induced apoptosis and extracellular matrix degradation by suppressing the NF‐kB and MAPK pathways in nucleus pulposus cells. Inflammation. 2017;40:725‐734. 28188410 10.1007/s10753-017-0516-6

[13] Deng X , Zhao F , Kang B , Zhang X . Elevated interleukin‐6 expression levels are associated with intervertebral disc degeneration. Exp Ther Med. 2016;11:1425‐1432. 27073460 10.3892/etm.2016.3079 PMC4812581

[14] Chen J , Xuan J , Gu YT , et al. Celastrol reduces IL‐1beta induced matrix catabolism, oxidative stress and inflammation in human nucleus pulposus cells and attenuates rat intervertebral disc degeneration in vivo. Biomed Pharmacother. 2017;91:208‐219. 28458159 10.1016/j.biopha.2017.04.093

[15] Yao Z , Nie L , Zhao Y , et al. Salubrinal suppresses IL‐17‐induced upregulation of MMP‐13 and extracellular matrix degradation through the NF‐kB pathway in human nucleus pulposus cells. Inflammation. 2016;39:1997‐2007. 27590238 10.1007/s10753-016-0435-y

[16] Wuertz K , Vo N , Kletsas D , Boos N . Inflammatory and catabolic signalling in intervertebral discs: the roles of NF‐κB and MAP kinases. Eur Cell Mater. 2012;23:103‐119: discussion 19‐20. 22354461 10.22203/ecm.v023a08

[17] Kang L , Hu J , Weng Y , Jia J , Zhang Y . Sirtuin 6 prevents matrix degradation through inhibition of the NF‐κB pathway in intervertebral disc degeneration. Exp Cell Res. 2017;352:322‐332. 28215636 10.1016/j.yexcr.2017.02.023

[18] Fujita N , Gogate SS , Chiba K , Toyama Y , Shapiro IM , Risbud MV . Prolyl hydroxylase 3 (PHD3) modulates catabolic effects of tumor necrosis factor‐alpha (TNF‐alpha) on cells of the nucleus pulposus through co‐activation of nuclear factor kappaB (NF‐κB)/p65 signaling. J Biol Chem. 2012;287:39942‐39953. 22948157 10.1074/jbc.M112.375964 PMC3501017

[19] Li J , Yuan W , Jiang S , et al. Prolyl‐4‐hydroxylase domain protein 2 controls NF‐κB/p65 transactivation and enhances the catabolic effects of inflammatory cytokines on cells of the nucleus pulposus. J Biol Chem. 2015;290:7195‐7207. 25635047 10.1074/jbc.M114.611483 PMC4358139

[20] Carbone C , Piro G , Merz V , et al. Angiopoietin‐like proteins in angiogenesis, inflammation and cancer. Int J Mol Sci. 2018;19:431. 10.3390/ijms19020431 PMC5855653 29389861

[21] Siddiqa A , Ahmad J , Ali A , Paracha RZ , Bibi Z , Aslam B . Structural characterization of ANGPTL8 (betatrophin) with its interacting partner lipoprotein lipase. Comput Biol Chem. 2016;61:210‐220. 26908254 10.1016/j.compbiolchem.2016.01.009

[22] Wang K , Liu W , Song Y , et al. The role of angiopoietin‐2 in nucleus pulposus cells during human intervertebral disc degeneration. Lab Invest. 2017;97:971‐982. 28394321 10.1038/labinvest.2017.35

[23] Abu‐Farha M , Abubaker J , Tuomilehto J . ANGPTL8 (betatrophin) role in diabetes and metabolic diseases. Diabetes Metab Res Rev. 2017;33:e2919. 10.1002/dmrr.2919 28722798

[24] Luo M , Peng D . ANGPTL8: an important regulator in metabolic disorders. Front Endocrinol (Lausanne). 2018;9:169. 29719529 10.3389/fendo.2018.00169 PMC5913278

[25] Rong Guo X , Li Wang X , Chen Y , et al. ANGPTL8/betatrophin alleviates insulin resistance via the Akt‐GSK3β or Akt‐FoxO1 pathway in HepG2 cells. Exp Cell Res. 2016;345:158‐167. 26387753 10.1016/j.yexcr.2015.09.012

[26] Yi P , Park JS , Melton DA . Betatrophin: a hormone that controls pancreatic beta cell proliferation. Cell. 2013;153:747‐758. 23623304 10.1016/j.cell.2013.04.008 PMC3756510

[27] Huang Y , Chen X , Chen X , et al. Angiopoietin‐like protein 8 in early pregnancy improves the prediction of gestational diabetes. Diabetologia. 2018;61:574‐580. 29167926 10.1007/s00125-017-4505-y

[28] Zhang Y , Guo X , Yan W , et al. ANGPTL8 negatively regulates NF‐κB activation by facilitating selective autophagic degradation of IKKgamma. Nat Commun. 2017;8:2164. 29255244 10.1038/s41467-017-02355-w PMC5735157

[29] Pfirrmann CW , Metzdorf A , Zanetti M , Hodler J , Boos N . Magnetic resonance classification of lumbar intervertebral disc degeneration. Spine. 2001;26:1873‐1878. 11568697 10.1097/00007632-200109010-00011

[30] Wu X , Song Y , Liu W , et al. IAPP modulates cellular autophagy, apoptosis, and extracellular matrix metabolism in human intervertebral disc cells. Cell Death Discov. 2017;3:16107. 28149534 10.1038/cddiscovery.2016.107 PMC5280875

[31] Han B , Zhu K , Li FC , et al. A simple disc degeneration model induced by percutaneous needle puncture in the rat tail. Spine. 2008;33:1925‐1934. 18708924 10.1097/BRS.0b013e31817c64a9

[32] Elliott DM , Yerramalli CS , Beckstein JC , Boxberger JI , Johannessen W , Vresilovic EJ . The effect of relative needle diameter in puncture and sham injection animal models of degeneration. Spine. 2008;33:588‐596. 18344851 10.1097/BRS.0b013e318166e0a2

[33] Mao HJ , Chen QX , Han B , et al. The effect of injection volume on disc degeneration in a rat tail model. Spine. 2011;36:E1062‐E1069. 21358491 10.1097/BRS.0b013e3182027d42

[34] Masuda K , Aota Y , Muehleman C , et al. A novel rabbit model of mild, reproducible disc degeneration by an anulus needle puncture: correlation between the degree of disc injury and radiological and histological appearances of disc degeneration. Spine. 2005;30:5‐5750. 15626974 10.1097/01.brs.0000148152.04401.20

[35] Wu X , Liu Y , Guo X , et al. Prolactin inhibits the progression of intervertebral disc degeneration through inactivation of the NF‐κB pathway in rats. Cell Death Dis. 2018;9:98. 29367664 10.1038/s41419-017-0151-z PMC5833353

[36] Kang L , Yang C , Song Y , et al. MicroRNA‐494 promotes apoptosis and extracellular matrix degradation in degenerative human nucleus pulposus cells. Oncotarget. 2017;8:27868‐27881. 28427186 10.18632/oncotarget.15838 PMC5438614

[37] Li J , Guan H , Liu H , et al. Epoxyeicosanoids prevent intervertebral disc degeneration in vitro and in vivo. Oncotarget. 2017;8:3781‐3797. 28052015 10.18632/oncotarget.14389 PMC5354795

[38] Wang WJ , Yu XH , Wang C , et al. MMPs and ADAMTSs in intervertebral disc degeneration. Clin Chim Acta. 2015;448:238‐246. 26162271 10.1016/j.cca.2015.06.023

[39] Erwin WM , DeSouza L , Funabashi M , et al. The biological basis of degenerative disc disease: proteomic and biomechanical analysis of the canine intervertebral disc. Arthritis Res Ther. 2015;17:240. 26341258 10.1186/s13075-015-0733-z PMC4560915

[40] Zhou X , Chen L , Grad S , et al. The roles and perspectives of microRNAs as biomarkers for intervertebral disc degeneration. J Tissue Eng Regen Med. 2017;11:3481‐3487. 28256798 10.1002/term.2261

[41] Wang SZ , Rui YF , Lu J , Wang C . Cell and molecular biology of intervertebral disc degeneration: current understanding and implications for potential therapeutic strategies. Cell Prolif. 2014;47:381‐390. 25112472 10.1111/cpr.12121 PMC6495969

[42] Kepler CK , Ponnappan RK , Tannoury CA , Risbud MV , Anderson DG . The molecular basis of intervertebral disc degeneration. Spine J. 2013;13:318‐330. 23537454 10.1016/j.spinee.2012.12.003

[43] Friedmann A , Goehre F , Ludtka C , et al. Microstructure analysis method for evaluating degenerated intervertebral disc tissue. Micron. 2017;92:51‐62. 27871028 10.1016/j.micron.2016.10.002

[44] Iatridis JC , Nicoll SB , Michalek AJ , Walter BA , Gupta MS . Role of biomechanics in intervertebral disc degeneration and regenerative therapies: what needs repairing in the disc and what are promising biomaterials for its repair? Spine J. 2013;13:243‐262. 23369494 10.1016/j.spinee.2012.12.002 PMC3612376

[45] Kadow T , Sowa G , Vo N , Kang JD . Molecular basis of intervertebral disc degeneration and herniations: what are the important translational questions? Clin Orthop Relat Res. 2015;473:1903‐1912. 25024024 10.1007/s11999-014-3774-8 PMC4418989

[46] Zhang F , Zhao X , Shen H , Zhang C . Molecular mechanisms of cell death in intervertebral disc degeneration (Review). Int J Mol Med. 2016;37:1439‐1448. 27121482 10.3892/ijmm.2016.2573 PMC4866972

[47] Sarath Babu N , Krishnan S , Brahmendra Swamy CV , Venkata Subbaiah GP , Gurava Reddy AV , Idris MM . Quantitative proteomic analysis of normal and degenerated human intervertebral disc. Spine J. 2016;16:989‐1000. 27125197 10.1016/j.spinee.2016.03.051

[48] Molinos M , Almeida CR , Caldeira J , Cunha C , Goncalves RM , Barbosa MA . Inflammation in intervertebral disc degeneration and regeneration. J R Soc Interface. 2015;12:20141191. 26040602 10.1098/rsif.2015.0429 PMC4528607

[49] Kang R , Li H , Rickers K , Ringgaard S , Xie L , Bunger C . Intervertebral disc degenerative changes after intradiscal injection of TNF‐alpha in a porcine model. Eur Spine J. 2015;24:2010‐2016. 25850392 10.1007/s00586-015-3926-x

[50] Weber KT , Alipui DO , Sison CP , et al. Serum levels of the proinflammatory cytokine interleukin‐6 vary based on diagnoses in individuals with lumbar intervertebral disc diseases. Arthritis Res Ther. 2016;18:3. 26743937 10.1186/s13075-015-0887-8 PMC4718017

[51] Li P , Gan Y , Xu Y , et al. 17beta‐estradiol attenuates TNF‐alpha‐induced premature senescence of nucleus pulposus cells through regulating the ROS/NF‐κB pathway. Int J Biol Sci. 2017;13:145‐156. 28255267 10.7150/ijbs.16770 PMC5332869

[52] Wang G , Huang K , Dong Y , et al. Lycorine suppresses endplate‐chondrocyte degeneration and prevents intervertebral disc degeneration by inhibiting NF‐κB signalling pathway. Cell Physiol Biochem. 2018;45:1252‐1269. 29448253 10.1159/000487457

[53] Mori N , Yamada Y , Ikeda S , et al. Bay 11–7082 inhibits transcription factor NF‐κB and induces apoptosis of HTLV‐I‐infected T‐cell lines and primary adult T‐cell leukemia cells. Blood. 2002;100:1828‐1834. 12176906 10.1182/blood-2002-01-0151

[54] Teixeira GQ , Leite Pereira C , Castro F , et al. Anti‐inflammatory Chitosan/Poly‐γ‐glutamic acid nanoparticles control inflammation while remodeling extracellular matrix in degenerated intervertebral disc. Acta Biomater. 2016;42:168‐179. 27321188 10.1016/j.actbio.2016.06.013

[55] Vadala G , Russo F , Di Martino A , Denaro V . Intervertebral disc regeneration: from the degenerative cascade to molecular therapy and tissue engineering. J Tissue Eng Regen Med. 2015;9:679‐690. 23512973 10.1002/term.1719

[56] Lu B , Moser A , Shigenaga JK , Grunfeld C , Feingold KR . The acute phase response stimulates the expression of angiopoietin like protein 4. Biochem Biophys Res Comm. 2010;391:1737‐1741. 20043872 10.1016/j.bbrc.2009.12.145

[57] Kanda A , Noda K , Oike Y , Ishida S . Angiopoietin‐like protein 2 mediates endotoxin‐induced acute inflammation in the eye. Lab Invest. 2012;92:1553‐1563. 22868908 10.1038/labinvest.2012.111

[58] Mysore R , Ortega FJ , Latorre J , et al. MicroRNA‐221‐3p regulates angiopoietin‐like 8 (ANGPTL8) expression in adipocytes. J Clin Endocrinol Metab. 2017;102:4001‐4012. 28938482 10.1210/jc.2017-00453

[59] Zhongyi S , Sai Z , Chao L , Jiwei T . Effects of nuclear factor kappa B signaling pathway in human intervertebral disc degeneration. Spine. 2015;40:224‐232. 25494317 10.1097/BRS.0000000000000733

[60] Purmessur D , Walter BA , Roughley PJ , Laudier DM , Hecht AC , Iatridis J . A role for TNFα in intervertebral disc degeneration: a non‐recoverable catabolic shift. Biochem Biophys Res Comm. 2013;433:151‐156. 23438440 10.1016/j.bbrc.2013.02.034 PMC3640343

