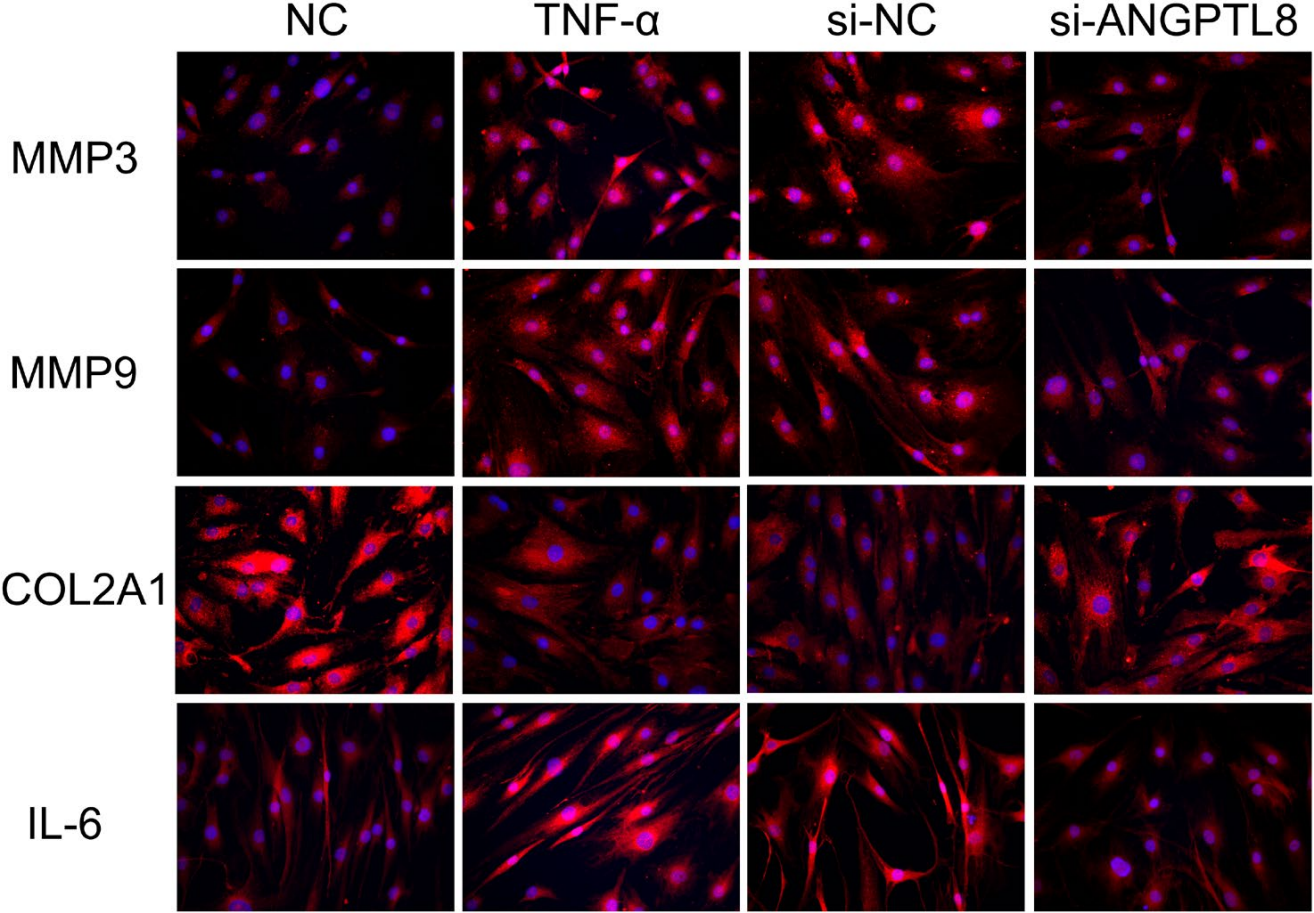# Corrigendum to “Angiopoietin‐Like Protein 8 Expression and Association With Extracellular Matrix Metabolism and Inflammation During Intervertebral Disc Degeneration”

**DOI:** 10.1111/jcmm.70646

**Published:** 2025-07-26

Z. Liao, X. Wu, Y. Song, et al., “Angiopoietin‐like protein 8 expression and association with extracellular matrix metabolism and inflammation during intervertebral disc degeneration”, *Journal of Cellular and Molecular Medicine*, 23 (2019): 5737–5750. https://doi.org/10.1111/jcmm.14488.

In the article, Figure 3N and Figure 5P contained errors due to duplication. The correct figures are shown below. The authors confirmed that these errors do not affect the overall conclusions of the study.